# Composite Films of Gelatin/Sodium Alginate Loaded with Benzyl Isothiocyanate and Eugenol Essential Oils: Characterization and Application in the Preservation of Cherries and Beef

**DOI:** 10.3390/foods15132327

**Published:** 2026-07-01

**Authors:** Siyi Bao, Jinle Ma, Jianan Liu, Hongman Hou, Jingran Bi, Xufen Xie, Hongshun Hao, Gongliang Zhang

**Affiliations:** 1 School of Food Science and Technology, Dalian Polytechnic University, Dalian 116034, China; bossiyi@163.com (S.B.); 18340658166@163.com (J.M.); liujianan_xg@163.com (J.L.); houhongman@dlpu.edu.cn (H.H.); bijingran1225@foxmail.com (J.B.); 2 Research Institute of Photonics, Dalian Polytechnic University, Dalian 116034, China; xiexufen@126.com; 3 Department of Inorganic Nonmetallic Materials Engineering, Dalian Polytechnic University, Dalian 116034, China; beike1952@163.com

**Keywords:** benzyl isothiocyanate, eugenol, gelatin, sodium alginate, composite film

## Abstract

In this study, two antimicrobial food packaging films were prepared by using gelatin/sodium alginate (GSA) as the film substrate and introducing benzyl isothiocyanate (BITC) and eugenol (EUG), respectively. The incorporation of BITC and EUG increased the tensile strength of the GSA film by 66.7% and 32.2%, respectively, while reducing the elongation at break by 44.9% and 39.8%, respectively. The water contact angle increased by 50.4% and 14.9%, and the water vapor permeability decreased by 65.4% and 59.2%, respectively, indicating that the addition of BITC and EUG improved the water resistance of the GSA film. In addition, the incorporation of BITC and EUG reduced the light transmittance of the GSA film. Scanning electron microscopy revealed that the surface inhomogeneity of the GSA film improved after the addition of BITC and EUG. Fourier-transform infrared spectroscopy (FTIR), X-ray diffraction (XRD), and thermogravimetric analysis (TGA) indicated that BITC and EUG interacted with the GSA matrix and affected the structural and thermal characteristics of the composite films. Application tests on cherries and beef have shown that BITC-GSA and EUG-GSA films delayed quality deterioration during storage. Overall, these films show promising potential as biodegradable active packaging materials for food preservation. These two films were applied to cherries and beef, effectively extending their shelf life and demonstrating the potential of these films as food packaging materials.

## 1. Introduction

Food packaging is a critical unit operation within food processing, serving essential functions in preserving quality and extending shelf life [1]. Owing to environmental concerns and consumer demand for natural, chemical-free preservatives and high-quality foods, biobased films enriched with natural compounds are receiving increased attention from the food packaging industry [2]. Owing to their excellent biodegradability, biobased films are considered potential replacements for plastic packaging. Moreover, the incorporation of antimicrobial agents (e.g., essential oils) endows the films with the ability to reduce microbiologically induced food spoilage, thus extending the shelf life of food products [3].

Among the numerous biopolymers, gelatin (G) and sodium alginate (SA) are widely recognized for their excellent film-forming capabilities, nontoxicity, biodegradability, and commercial availability. Gelatin, a water-soluble protein derived from the partial hydrolysis of collagen, adopts a random coil conformation in aqueous solutions [4]. G consists of various amino acids, and the presence of aromatic residues enables it to readily absorb UV light, thereby helping to delay chemical oxidation in food systems [5]. SA is a straight-chain polysaccharide consisting of *β*-D-mannuronic acid and *α*-D-mannuronic acid linked by 1-*β*-4 glycosidic bonds [6]. SA is not only inexpensive and nontoxic, but also offers advantages in terms of mechanical properties when it is used to form biodegradable films. Both G and SA are hydrophilic biopolymers with superior affinity and compatibility, so they are expected to form composite films with better properties. It has been reported that a large number of reactive groups on G with −COOH and −NH_2_ could interact with the hydroxyl group (−OH) on SA to improve the mechanical properties of the composite film [7]. Therefore, blending can unite the benefits of these two biopolymers.

In recent years, some active compounds (e.g., essential oils and polyphenols) have been added to food packaging materials to confer antimicrobial and antioxidant capabilities, slow the oxidation of fats in food, and inhibit microbial growth, thus prolonging the shelf-life of food. Karami et al. [8] added yarrow essential oil to gelatin/sodium alginate (GSA) film, effectively inhibiting the growth of *Pseudomonas aeruginosa* (*P. aeruginosa*) and *Escherichia coli* (*E. coli*). Moreover, Charri et al. [9] reported that GSA film alone did not have any inhibitory effect on *Staphylococcus aureus* (*S. aureus*), *Listeria monocytogenes* (*L. monocytogenes*), or *E. coli*, and when beetroot extract was added to the GSA film, it showed greater antimicrobial activity. Loading of GSA film with tea polyphenols conferred antioxidant capacity [10]. However, as G and SA exhibit limited antimicrobial activity, the antimicrobial properties of the GSA film are relatively weak. The incorporation of natural antimicrobial agents into the GSA film thus provides an effective strategy to enhance the antimicrobial performance of the composite films.

Benzyl isothiocyanate (BITC), a member of the Cruciferae family of isothiocyanates (ITCs), is a natural food preservative recognized as safe. Many studies have shown that BITC has antimicrobial activity against various foodborne pathogens, including *E. coli*, *S. aureus*, *Salmonella typhimurium* (*S. typhimurium*), and *L. monocytogenes* [11]. To improve the antimicrobial capacity of a *κ*-carrageenan (KC) based film, Huang et al. [12] incorporated BITC-*β*-cyclodextrin (BITC-*β*-CD) into it. The BITC-*β*-CD-KC film was shown to positively inhibit both *S. aureus* and *L. monocytogenes*. Moreover, Wu et al. [13] loaded BITC-*α*-cyclodextrin (BITC-*α*-CD) into chitosan films, which further improved the growth inhibition rate of chitosan films against *S. typhimurium*, and effectively prolonged the shelf-life of beef. Eugenol (EUG) is a natural phenolic compound that has been widely used in active packaging materials because of its powerful antimicrobial and antioxidant properties. It has been reported that EUG can be incorporated into zein and poly (lactic acid) film blends to provide antimicrobial activity to blended films [14]. EUG was doped into a gelatin/chitosan film, and the elasticity of the film increased, significantly improving its oxidation resistance [15]. However, most reported essential oil-based biodegradable films focus on specific polymer matrices, encapsulation strategies, or single food preservation systems. Limited information is available on the material properties, structural characteristics, and preservation performance of GSA composite films containing BITC or EUG. Therefore, further evaluation of these films is needed to clarify their potential application as biodegradable active packaging materials.

Accordingly, GSA-based active films containing BITC or EUG were developed in this work. The physicochemical and functional properties of the films, including mechanical performance, light barrier ability, water vapor barrier properties, and surface wettability, were evaluated. Fourier-transform infrared spectroscopy (FTIR), X-ray diffraction (XRD), thermogravimetric analysis (TGA), and scanning electron microscopy (SEM) were used to characterize the structural, thermal stability, and morphological features of the films. Finally, cherries and beef were selected as representative food models to evaluate the preservation performance of the developed films during storage.

## 2. Materials and Methods

### 2.1. Materials

BITC (98%), EUG (98%), G, and SA were acquired from Aladdin, Inc. (Shanghai, China). Glycerol was purchased from Tianjin Damao Chemical Reagent Factory (Tianjin, China). Sodium chloride, magnesium oxide, hydrochloric acid, orthoboric acid, and sodium hydroxide were purchased from Shanghai Macklin Biochemical Technology Co., Ltd. (Shanghai, China). Plate count agar was obtained from Qingdao Hope Bio-Technology Co., Ltd. (Qingdao, China). 2-Thiobarbituric acid was sourced from Sangon Biotech (Shanghai) Co., Ltd. (Shanghai, China). All other reagents are of analytical grade.

### 2.2. Preparation of Composite Films

The gelatin powder was dissolved in distilled water and stirred at 60 °C for 15 min to obtain a 1% gelatin solution. Sodium alginate powder (3 g) was dissolved in the gelatin solution and stirred at 45 °C for 20 min. Glycerin (1% *v*/*v*) was then added as a plasticizer. Finally, BITC or EUG was added individually at 0.2% *v*/*v* relative to the total solution to prepare BITC-GSA and EUG-GSA film-forming solutions, respectively. The film solution (20 mL) was poured into a disposable culture dish (90 mm diameter) and dried in an oven at 45 °C for 12 h until a constant weight was reached. After drying, the films were equilibrated at room temperature under 50% relative humidity for 12 h before further use.

### 2.3. Characterization of Composite Films

#### 2.3.1. Thickness and Mechanical Properties

The prepared films were cut into strips measuring 40 mm × 10 mm, and the thickness of each film was measured at multiple positions using a thickness gauge (32CHF2530, Deqing Shengtai Xin Electronics Technology Co., Ltd., Deqing, China). The tensile strength and elongation at break were determined using an Instron 5982 universal testing machine (Instron Corporation, Norwood, MA, USA) at a crosshead speed of 5 cm/min until sample failure. Each film sample was tested in triplicate to evaluate its mechanical properties.

#### 2.3.2. Water Vapor Permeability (WVP)

WVP was determined according to the ASTM E96−95 (1995) [16] with minor modifications [17]. The film was cut into 60 mm circular pieces, sealed on the top of a weighing flask containing 20 mL of saturated sodium chloride solution (75% RH), and placed in a desiccator containing fully dried calcium chloride (0% RH). The flasks were weighed at 24 h intervals for three consecutive days, and the WVP was calculated according to the following Formula (1):

(1) $$WVP(g/m\cdot s\cdot Pa)=\frac{\Delta m\cdot x}{A\cdot \Delta t\cdot \Delta p}$$

where Δ*m* represents the mass gained (g), *x* is the average thickness of the film (mm), *A* is the area of the film through which water vapor permeates (m^2^), Δ*t* is the measurement time (d), and Δ*p* is the difference in water vapor pressure across the film (2.809 kPa at 20 °C).

#### 2.3.3. Water Contact Angle (WCA)

The WCA of the film was determined using a contact angle tester (Kruss DSA 25, Kruss Ltd., Hamburg, Germany), with 5 µL of ultrapure water deposited on the film surface using a micro syringe, and images were captured at room temperature. Each film was measured five times.

#### 2.3.4. Transmittance and Opacity

The films were punched into pieces matching the size of the wells of a microplate and placed into the wells. A microplate reader (SpectraMax M2, Molecular Devices Ltd., Silicon Valley, CA, USA) was used to analyze the transmittance of the film in the wavelength range of 200–800 nm, with each measurement performed in triplicate.

A UV–visible spectrophotometer (UV-1750, Shimadzu Co., Ltd., Kyoto, Japan) was used to determine the opacity of the films. The film samples were cut into 1 cm × 3 cm pieces and affixed onto the surface of a cuvette. Each sample was measured in triplicate, and the opacity was calculated using the following Equation (2):

(2) $$opacity=\frac{A_{600\;\mathrm{nm}}}{x}$$

where *A*_600 nm_ is the absorption value at 600 nm and *x* is the film thickness (mm).

#### 2.3.5. Color

The color of the film was measured by a colorimeter (DS-700D, Shenzhen Jinzhun Instrument Equipment Co., Ltd., Shenzhen, China). The instrument was calibrated using a standard whiteboard as a measurement background. The apparent color of the film was evaluated by L* (black/white), a* (green/red), and b* (blue/yellow) at multiple positions on the film surface at room temperature, with each measurement performed in triplicate.

### 2.4. Structural and Thermal Characterization of the Composite Films

#### 2.4.1. FTIR

A mixture for FTIR measurement was prepared by homogenizing the dried and pulverized film with KBr at a 1:100 ratio. FTIR spectroscopy was then performed using an FTIR spectrometer (Nicolet iS20, Thermo Fisher Scientific Inc., Waltham, MA, USA) in the range of 4000–500 cm^−1^ with a resolution of 4 cm^−1^. Each sample was scanned 32 times cumulatively, and baseline correction was performed using an air background.

#### 2.4.2. X-Ray Diffraction (XRD)

X-ray diffraction (XRD) analysis of the film samples was performed using an X-ray diffractometer (Rigaku Ultima IV, Rigaku Co., Ltd., Tokyo, Japan). The operating voltage and current were set at 40 kV and 40 mA, respectively. The samples were scanned over a 2θ range of 5–60° at a scanning rate of 5°/min.

#### 2.4.3. Thermal Stability

Thermogravimetric analysis (TGA) of the film samples was performed using a thermogravimetric analyzer (STA-8000, PerkinElmer Co., Ltd., Waltham, MA, USA). Approximately 5 mg of each sample was heated from room temperature to 600 °C at a heating rate of 10 °C/min under a nitrogen atmosphere, and the changes in sample weight and weight-loss rate as a function of temperature were recorded.

#### 2.4.4. SEM

The film samples were fixed to the sample stage with conductive adhesive tape and coated with a thin layer of gold by sputter coating. Liquid nitrogen was used to brittle-fracture the films to obtain cross-sections. SEM (Quanta 450, FEI Company Hillsboro, OR, USA) was used to observe the surface and cross-section of the films at an accelerating voltage of 10 kV.

### 2.5. Experiments on the Preservation of Cherries and Chilled Beef by Composite Films

#### 2.5.1. Composite Films for the Preservation of Cherries

Cherries were used as a fruit model to evaluate the effects of different films on fruit preservation. The fruits were surface-sterilized by immersion in 0.3% sodium hypochlorite solution for 3 min and subsequently rinsed with sterile water to ensure uniform initial conditions. Fifteen cherries were randomly selected and divided into five groups: the control group (without any film wrapping), polyethylene (PE) film, GSA film, BITC-GSA and EUG-GSA film. Each cherry was individually wrapped with the assigned film and stored at 4 °C and 70% relative humidity. Physiological indices were measured at 3-day intervals over a 15-day storage period. During storage, representative cherries from each group were photographed under the same lighting conditions and at the same distance to record changes in appearance, including surface shrinkage, browning, and decay.

The decay rate was assessed on the basis of the area of the affected fruit, and fruits for which less than four-fifths of the edible portion remained were considered decayed [17]. The decay rate is given by the following Formula (3):

(3) $$Decay\;rate=\frac{Number\;of\;decayed\;cherries}{Total\;number\;of\;cherries}$$

Weight loss (%) was defined as the initial weight of cherries (W_0_) minus the weight of cherries at different storage times (W_1_) and was calculated as follows (4):

(4) $$Weight\;loss\left(\%\right)=\frac{W_{0}-W_{1}}{W_{0}}\times 100$$

The hardness of cherries at different storage times was determined using a texture analyzer (TA. XT Plus C, Stable Micro Systems Ltd., Godalming, Surrey, UK) with a needle probe. The probe was inserted vertically into the fruit at a speed of 1 mm/s to a depth of 6 mm. A total of 5 g of cherries was accurately weighed and ground into a homogenate, and centrifuged, and the supernatant was used for the pH assay. The pH of the cherry juice was measured using a pH meter (G1200328N, Mettler-Toledo Instruments Co., Ltd., Shanghai, China) calibrated with standard buffer solutions, with the probe immersed directly in the supernatant to obtain readings. The content of soluble solids was determined by using a handheld refractometer (IR240, Maiyi Technology Co., Ltd., Shanghai, China). Titratable acid was measured using 5 g of cherry homogenate titrated to pH 8.1 with 0.1 mol/L NaOH and given as a percentage of citric acid [18].

#### 2.5.2. Composite Films for Beef Preservation

Fresh beef samples were cut into small square pieces (approximately 3 g) with a sterile knife. Each piece of beef was individually wrapped with a single film sample. Four groups were established—PE film, GSA film, BITC-GSA film, and EUG-GSA film—with three replicates per group. All the samples were then stored at 4 °C and 70% relative humidity for 14 days, and removed at two-day intervals for analysis.

For pH determination, 3 g of beef was homogenized with 30 mL of distilled water and shaken for 30 min. The mixture was then filtered, and the pH of the filtrate was measured using a pH meter (G1200328N; Mettler-Toledo Instruments Co., Ltd., Shanghai, China). Each measurement was performed in triplicate.

Lipid oxidation was assessed by measuring thiobarbituric acid reactive substances (TBARS). Minced beef was mixed with 20% trichloroacetic acid and homogenized at 6000 rpm for 2 min. After centrifugation, the supernatant was mixed in equal volume with 0.02 mol/L 2-thiobarbituric acid solution and incubated in a boiling water bath for 20 min. After cooling, the absorbance at 532 nm was measured using a microplate reader, and the TBA concentration was calculated as the malondialdehyde (MDA) concentration (mg/kg) from a standard curve.

Total volatile basic nitrogen (TVB-N) and total viable count (TVC) were determined according to the Chinese national standards GB 5009.228-2016 and GB 4789.2-2016, respectively [19,20]. The sensory quality of the beef samples was evaluated according to a previously reported method with slight modifications. Ten trained assessors scored the samples for color, viscosity, odor, visible impurities, and texture using a 10-point scale, where 10 represented the best quality, 5 indicated the acceptable limit, and 1 represented the worst quality. All assays were conducted in triplicate for each treatment group.

### 2.6. Statistical Analysis

The experimental data are expressed as the mean ± standard deviation and were measured in triplicate (*n* = 3). Figures, principal component analysis (PCA), and Pearson correlation analysis were generated using Origin Pro 2019b software. PCA was performed on the basis of the correlation matrix to reduce the influence of differences in units and scales among variables. A one-way ANOVA with Tukey’s HSD test was performed at the 95% confidence level using IBM SPSS 27.

## 3. Results and Discussion

### 3.1. Mechanical Properties of the GSA, BITC-GSA and EUG-GSA Films

**Figure 1:**
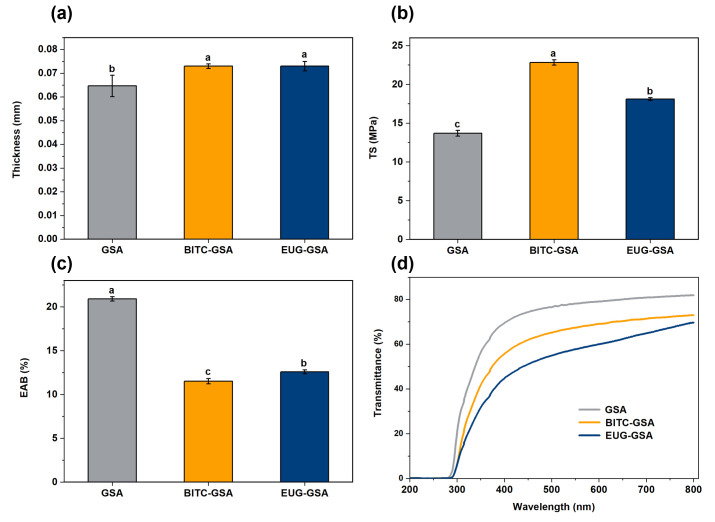
Thickness, mechanical properties and light transmittance of GSA films with and without BITC and EUG. (**a**) Thickness; (**b**) tensile strength; (**c**) elongation at break; (**d**) transmittance. Different lowercase letters indicate that the difference was statistically significant (*p* < 0.05).

The thickness, tensile strength (TS), and elongation at break (EAB) of the GSA, BITC-GSA, and EUG-GSA films are shown in Figure 1. The addition of EUG and BITC had comparable effects on the thickness of the GSA film, with each increasing by approximately 12.3% relative to that of the control sample (Figure 1a). Previous studies have suggested that the increase in film thickness is associated mainly with the accumulation of insoluble solids, rather than the specific type of essential oil incorporated into the film matrix [21]. Mechanical properties are important parameters for determining the structural integrity of a film. TS is an indicator of film strength, and EAB can reflect the flexibility of the film [22]. As shown in Figure 1b, compared with the GSA film (13.7 ± 0.4), the BITC-GSA (22.8 ± 0.3) and EUG-GSA (18.1 ± 0.2) films exhibited significantly greater tensile strength (TS). Bhatia et al. [23] reported that pectin-sodium alginate films loaded with Cassia essential oil also showed an increase in TS, possibly because of the cross-linking effect of the Cassia essential oil, which reduced the fluidity of the polymer matrix. In contrast, the EAB showed the opposite trend (Figure 1c). The EAB of the GSA film is 20.9 ± 0.3%, whereas that of the composite films significantly decreases, with EAB values of 11.5 ± 0.3% for BITC-GSA and 12.6 ± 0.2% for EUG-GSA. A similar study reported that the addition of tea polyphenols to GSA films resulted in an increase in TS and a decrease in EAB with increasing concentrations of tea polyphenols [24]. In general, the incorporation of hydrophobic compounds alters the mechanical behavior of films by reducing their flowability and durability, increasing their stiffness, and decreasing their flexibility [25]. Consistently, the introduction of BITC or EUG led to increased thickness and hardness in the GSA film, accompanied by a reduction in flexibility.

### 3.2. Transmittance of GSA, BITC-GSA, and EUG-GSA Films

Light barrier properties are important for food packaging films because light exposure can accelerate lipid oxidation, pigment degradation, vitamin loss, and sensory deterioration [2]. As shown in Figure 1d, the GSA, BITC-GSA, and EUG-GSA films exhibited effective ultraviolet light barrier capabilities with transmittances below 10% in the 200–280 nm range. In addition, in the visible range (350–800 nm), the transmittance of the BITC-GSA and EUG-GSA films was lower than that of the GSA film, with the EUG-GSA film having the lowest transmittance. The reduced light transmittance may be related to the aromatic structures of BITC and EUG, which can contribute to light absorption, as well as the dispersion of active compounds within the film matrix, which may increase light scattering and reduce light transmission [26]. Haghighi et al. loaded different plant essential oils into chitosan-gelatin films and reported that the light transmission ability of the essential oil-loaded film was lower than that of the pure chitosan-gelatin film [2]. Light barriers are important for preventing food spoilage, preventing the photooxidation of organic compounds, and preventing the degradation of vitamins and other pigments [27]. These results indicate that BITC and EUG enhanced the light-barrier performance of GSA films, which may be beneficial for reducing photo-oxidation and maintaining the quality of light-sensitive foods during storage.

### 3.3. Water Resistance of GSA, BITC-GSA, and EUG-GSA Films

**Figure 2:**
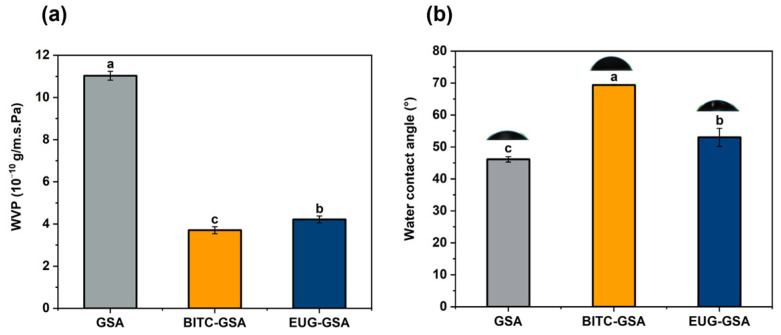
Water resistance of GSA films with and without BITC and EUG. (**a**) Water vapor permeability; (**b**) water contact angle. Different lowercase letters indicate that the difference was statistically significant (*p* < 0.05).

Avoiding or reducing moisture transfer between food and the surrounding atmosphere is a very important function of food packaging materials. Therefore, a low WVP favors the extension of food shelf life [1]. As depicted in Figure 2a, the GSA film had the highest WVP of 10.6 × 10^−10^ g/m·s·Pa. This may be because the GSA film is a polysaccharide–protein comingled film that is inherently hydrophilic [28]. With the addition of BITC and EUG, the WVP of the film was 3.7 × 10^−10^ g/m·s·Pa (BITC-GSA) and 4.45 × 10^−10^ g/m·s·Pa (EUG-GSA), respectively, which were significantly lower than those of the GSA film. The level of WVP depends on the proportion of hydrophilic/hydrophobic film [29]. This reduction may be related to the hydrophobic characteristics of BITC and EUG, which could decrease the affinity of the film matrix for water molecules and limit water vapor diffusion through the film. In addition, the incorporation of BITC and EUG may modify the internal diffusion pathway of water vapor within the composite matrix, thereby contributing to the reduced WVP [30]. Similar decreases in WVP have been reported in essential oil-incorporated polysaccharide-based films, such as cinnamon essential oil-loaded pectin–sodium alginate composite films [23].Therefore, the improved water vapor barrier properties of BITC-GSA and EUG-GSA films may be beneficial for reducing moisture transfer and maintaining food quality during storage.

The hydrophobicity of the film surface was evaluated using WCA. When the WCA is less than 90°, the film surface is hydrophilic and vice versa [12]. As indicated in Figure 2b, the WCA of the GSA film was lower at 46.1°. According to previous studies, the lower WCA of the GSA film is due to the ability of water to bind to the reactive groups in glycerol and gelatin [8]. With the addition of BITC and EUG, the WCA of the GSA film increased, which may have been due to the hydrophobicity of both, resulting in improved hydrophilicity of the film. Similar studies have reported that incorporating peppermint essential oil increases the surface hydrophobicity of gelatin-based edible films [31]. Overall, these results indicate that the incorporation of BITC and EUG enhanced the water barrier properties of the GSA film, thereby improving its resistance to moisture transmission.

### 3.4. Color and Opacity of GSA, BITC-GSA and EUG-GSA Films

The color of a packaging film directly influences the appearance and consumer acceptance of the product [32]. As summarized in Table 1, the incorporation of BITC and EUG significantly reduced the L* value of the GSA film to 59.1 and 44.0, respectively, indicating a darkening effect. Concurrently, a significant increase in the b* value was observed, suggesting an increase in yellowness. This phenomenon was more pronounced in the EUG-GSA film, which can likely be attributed to the intrinsically darker color of eugenol than that of BITC. These changes were visually confirmed in the high-resolution images of the films (Table 1), where compared with the BITC-GSA and pure GSA films, the EUG-GSA film appeared notably darker and more yellow. Moreover, the opacity of the GSA film increased significantly after the addition of BITC and EUG. The results of this experiment are possibly due to the interference of light transmission by light scattering from the oil droplets in the GSA film network [22]. Previous reports have demonstrated that high-opacity packaging materials are effective at preventing light-induced oxidation of food products [33]. Consistent with this, the incorporation of BITC and EUG increased the opacity of the GSA film, making it more suitable for food packaging purposes.

### 3.5. Microstructure of the GSA, BITC-GSA and EUG-GSA Films

**Figure 3:**
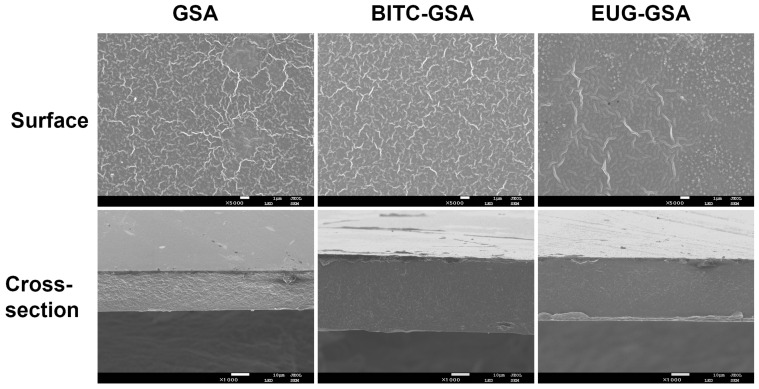
SEM images of the GSA film surface and cross-section with and without BITC and EUG. Surface (5000× magnification); cross-section (1000× magnification).

SEM analysis revealed the dispersion of different phases in the mixed system and the interactions at the interfaces, which can be used as indicators to assess the compatibility of the components in the matrix [34]. SEM images of the surface and cross-section of the three films are shown in Figure 3. Compared with those of the GSA film, the surfaces of the BITC-GSA and EUG-GSA films were smoother and more uniform. Notably, the EUG-GSA film displayed a marked reduction in surface wrinkles, which may be attributed to improved compatibility and stronger interactions between EUG and the GSA matrix, facilitating more homogeneous network formation.

The cross-sectional SEM images revealed that the incorporation of BITC and EUG led to a significant increase in film thickness, which is consistent with the observed mechanical property changes. Moreover, the cross-sections of the BITC-GSA and EUG-GSA films appeared denser and more compact than those of the GSA film, with no visible fractures or voids, suggesting enhanced structural integrity. These microstructural modifications support the conclusion that the addition of BITC and EUG not only reinforces the film matrix but also contributes to improved surface uniformity and overall film quality.

### 3.6. FTIR of the GSA, BITC-GSA, and EUG-GSA Films

**Figure 4:**
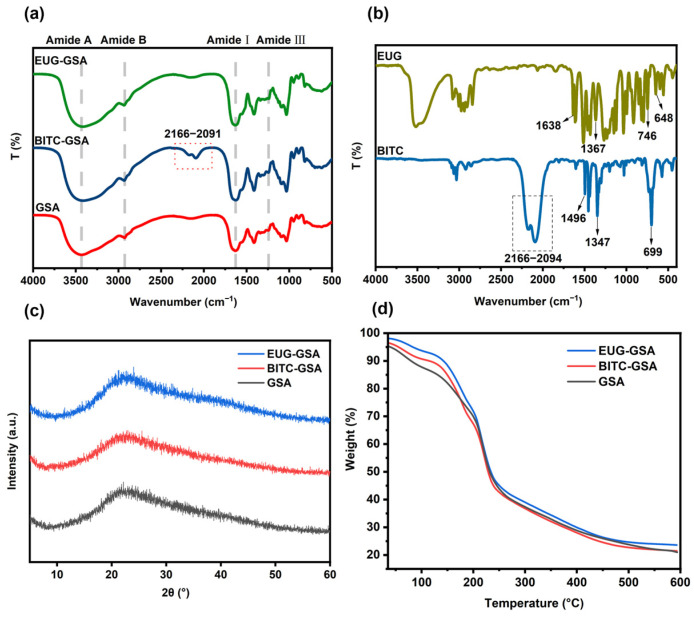
Structural and thermal characterization of GSA-based films: (**a**) FTIR spectra of GSA, BITC-GSA, and EUG-GSA films; (**b**) FTIR spectra of BITC and EUG; (**c**) XRD patterns of GSA, BITC-GSA, and EUG-GSA films; and (**d**) TGA thermograms of GSA, BITC-GSA, and EUG-GSA films.

The FTIR spectra of the GSA, BITC-GSA and EUG-GSA films are shown in Figure 4a. The characteristic absorption peaks of BITC at 2166–2094 cm^−1^ correspond to the stretching vibrations of the N=C=S group, whereas the sharp peaks at 1496 cm^−1^ and 1347 cm^−1^ arise from the vibrations of the carbon skeleton in the benzene ring, and the out-of-plane bending vibrational peak at 699 cm^−1^ is associated with C–H vibrations of the benzene ring [25]. EUG exhibited absorption peaks at 1637 cm^−1^, 1367 cm^−1^, 746 cm^−1^, and 647 cm^−1^, which were related mainly to phenyl groups [35]. In the GSA film, characteristic absorption bands were observed at 3434 cm^−1^ (amide-A) because of N–H and O–H stretching vibrations, 2928 cm^−1^ (amide-B) corresponding to C-H and -NH_2_ stretching, 1628 cm^−1^ (amide-I) arising from C=O stretching vibrations, and 1242 cm^−1^ (amide-III) assigned to the in-plane vibrations of the C–N and N–H bonds of the amide, and the CH_2_ group of glycine [36]. After the addition of BITC or EUG to the GSA film matrix, slight shifts in some absorption bands were observed. For example, the O-H stretching band at 3434 cm^−1^ in the pure GSA film shifted to 3417 cm^−1^ and 3414 cm^−1^ in the BITC-GSA and EUG-GSA films, respectively. A downward shift of the -OH absorption band was also found when yarrow essential oil was doped into the GSA film [8]. In addition, the characteristic absorption peak at 1628 cm^−1^ in the GSA film shifted to 1633 cm^−1^ and 1631 cm^−1^ in the BITC-GSA and EUG-GSA films, respectively, suggesting potential hydrogen bonding interactions between the essential oils and the GSA film. These results indicate that certain molecular forces, such as hydrogen bonding and cross-linking, are formed between the essential oils and the GSA film. Moreover, in the BITC-GSA and EUG-GSA films, some characteristic peaks of BITC and EUG were no longer observed in the spectra. For example, the benzene ring peak of BITC disappeared in the BITC-GSA film, whereas the N=C=S stretching peak remained. Similarly, the characteristic phenyl peaks of EUG were absent in the EUG-GSA film. These changes suggest that interactions occurred between these compounds and the GSA matrix, potentially involving embedding or strong intermolecular interactions.

### 3.7. XRD of GSA, BITC-GSA, and EUG-GSA Films

The crystalline structure and internal molecular arrangement of the films were further characterized by XRD analysis. As shown in Figure 4c, all films exhibited broad diffraction peaks at approximately 2θ = 20–25° and no obvious sharp crystalline peaks, indicating that GSA, BITC-GSA, and EUG-GSA mainly exhibited amorphous structures with limited crystallinity. No new diffraction peaks appeared after BITC or EUG incorporation, suggesting that the active compounds did not form independent crystalline phases in the film matrix. Similar phenomena have been reported in other essential oil-incorporated biopolymer films, in which the addition of peppermint essential oil did not significantly change the main amorphous characteristics of the carrageenan–konjac glucan composite film matrix [37]. Overall, the XRD results revealed that BITC and EUG incorporation did not induce new crystalline phases in the GSA-based films, and that the films remained predominantly amorphous.

### 3.8. Thermal Stability of GSA, BITC-GSA, and EUG-GSA Films

To evaluate the thermal stability of the composite films, TGA was performed by monitoring the weight changes of the films with increasing temperature. As shown in Figure 4d, the GSA, BITC-GSA, and EUG-GSA films exhibited a three-stage thermal degradation behavior. The first stage below approximately 120 °C was related mainly to the evaporation of water in the film matrix [38]. The second stage occurred at approximately 120–250 °C, during which time the weight loss became more pronounced, especially at approximately 220–250 °C. This stage was associated mainly with the volatilization or decomposition of small molecules, such as glycerol and active compounds, as well as the thermal degradation of gelatin and sodium alginate chains [39]. Above approximately 250 °C, the weight loss gradually increased, corresponding to the further decomposition of carbonaceous residues [40]. At 600 °C, the residual weights of GSA, BITC-GSA, and EUG-GSA were 21.0%, 21.5%, and 23.6%, respectively, indicating that BITC and EUG slightly increased the final thermal residue, particularly EUG. A similar phenomenon has been reported for oxidized corn starch/pullulan composite films, in which the incorporation of cinnamon essential oil nanoemulsions increased the final residue and improved the thermal behavior of the film matrix [41].

The FTIR, XRD, and TGA results indicated that BITC and EUG interacted with the GSA matrix and affected the internal structure and thermal behavior of the composite films. In addition, the antibacterial activity, biodegradability, and release behavior of BITC/EUG-loaded GSA films were evaluated in our previous study. The results revealed that the incorporation of BITC and EUG resulted in GSA films with strong antibacterial activity, a degradation rate above 80% after 28 days in the soil burial test, and a gradual release behavior of active compounds from the film matrix [42]. Based on these structural and functional foundations, cherries and beef were selected as food models to further evaluate the preservation performance of the films.

### 3.9. Freshness Preservation of Cherries by BITC-GSA and EUG-GSA Films

**Figure 5:**
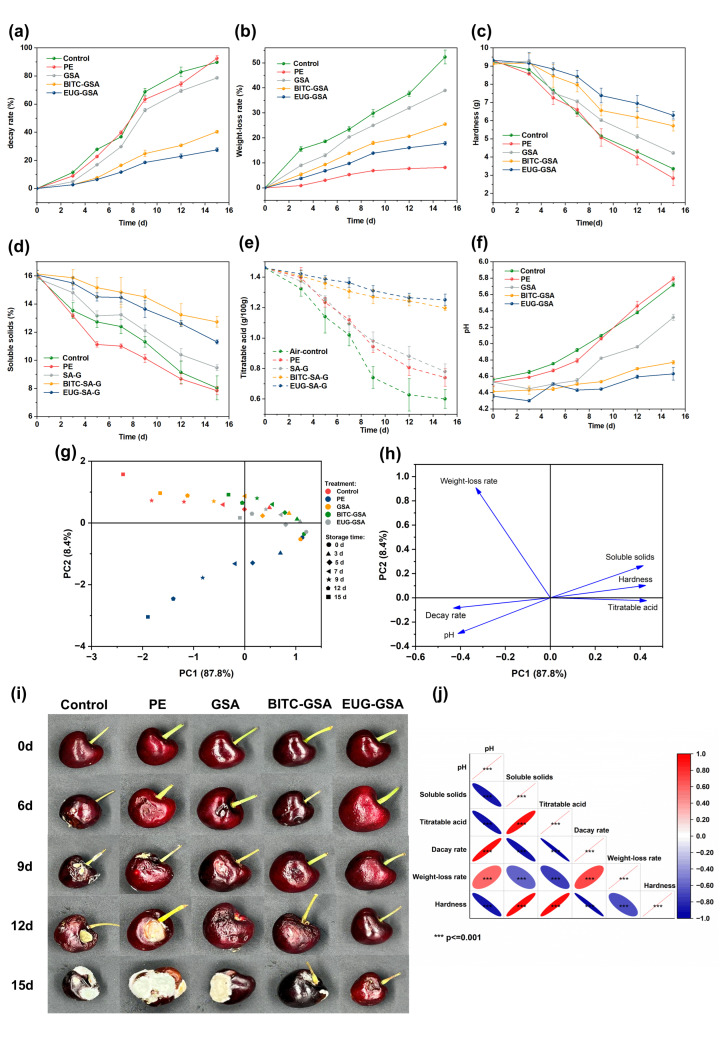
Effects of different films on cherry preservation: (**a**) decay rate; (**b**) weight loss rate; (**c**) hardness; (**d**) soluble solids; (**e**) titratable acid content; (**f**) pH value; (**g**) PCA score plot; (**h**) PCA loading plot; (**i**) appearance changes; and (**j**) correlation heatmaps of cherry quality indicators.

The fruit decay rate serves as a critical indicator for evaluating cherry freshness. As illustrated in Figure 5a, the decay rate increased over the storage period across all treatment groups. The control and PE film groups exhibited the highest decay rates, reaching 89.7% and 92.5%, respectively, by day 15. In contrast, cherries packaged with the EUG-GSA composite film demonstrated the most effective suppression of decay, maintaining the lowest incidence throughout the storage time. On day 15, the decay rate of the EUG-GSA film (27.5%) was only one-third that of the PE film. Although the BITC-GSA film did not inhibit the decay rate of cherry as much as the EUG-GSA film did, it was still much lower than the PE film. As a result, the BITC-GSA and EUG-GSA films were significantly better than commercially available cling film at preserving cherry freshness. The reduced decay rate in the BITC-GSA and EUG-GSA groups may be attributed to the combined effects of the active compounds and the film matrix. BITC and EUG have been reported to possess good antimicrobial activity, which may help inhibit the microbial growth associated with fruit decay [43]. In a similar study, sodium alginate/glucomannan-activated films containing lycopene microcapsules reduced cherry decay because of the favorable bacterial inhibition by chitosan and carboxymethyl chitosan and the antioxidant effect of lycopene [17].

The weight loss rate serves as an indicator of changes in fruit weight during storage. As shown in Figure 5b, weight loss increased in all treatment groups during storage, primarily because of the consumption of stored nutrients and respiratory activity associated with fruit metabolism. The highest rate of weight loss was recorded in the control group (52.4%), followed by the GSA film group (38.9%). Interestingly, PE resulted in the lowest weight loss, which increased from 0% to 8.2% during storage. This phenomenon was caused by the high barrier properties of the PE film, which resulted in low weight loss, but its high decay rate could not be ignored. The weight loss of the EUG-GSA film was only 17.8% on day 15, indicating good quality assurance. In addition, the weight loss of the BITC-GSA film was significantly lower. This result was attributed to the dense structure of the GSA composite film matrix, which effectively hindered water migration, inhibited cherry respiration, and slowed the increase in the weight loss rate. Moreover, BITC and EUG have excellent antimicrobial effects, preventing cherries from experiencing decay and water loss because of bacterial contamination.

The variation in hardness of cherries is shown in Figure 5c. On day 8, the hardness of cherries in the PE group decreased noticeably. Although PE film may reduce water loss, its relatively low permeability could restrict gas exchange and promote moisture accumulation. In addition, the visual images recorded on days 6 and 9 revealed obvious quality deterioration in the PE group (Figure 5i), suggesting that the hardness loss during this period may be associated with physiological softening, moisture accumulation, restricted gas exchange, and visible decay development. The final hardness values of cherries wrapped with BITC-GSA and EUG-GSA films were 2.9 g and 3.5 g greater than that of those wrapped with PE, respectively. The GSA film, especially that containing EUG, can effectively prevent a decrease in cherry hardness, maintain the hardness of cherries, and prolong the shelf-life. Similar studies have reported that carboxymethyl cellulose/gelatin composite films incorporating zein-stabilized lemon essential oil Pickering emulsions effectively maintain the firmness of cherries [44].

The variation in soluble solids content reflects the change in quality and the degree of ripening of the fruit during storage. The variation in the soluble solids content of cherries during different storage periods is shown in Figure 5d. The starting content of soluble solids in cherries is approximately 16%. Over time, the respiration of cherries led to an increase in nutrient depletion and an overall decreasing trend in soluble solids during storage. Cherries packed in PE film had the lowest soluble solids content, which may be related to the higher decay rate of cherries and the consumption of sugars by microorganisms [45]. The soluble solids content of cherries wrapped in BITC-GSA and EUG-GSA films slowly decreased from 16% to 12.7% and 11.3%, respectively, within 15 days of storage compared with that of the control. It was speculated that the BITC-GSA and EUG-GSA films might have inhibited the respiration and metabolism of the cherries, thus slowing the consumption of sugars and prolonging the preservation time of the cherries. In addition, the low-temperature environment was also an important factor leading to the reduction in cherry respiration.

The titratable acid content of cherries tended to decrease during storage, probably because of the consumption of organic acids when respiration occurs in cherries, thus decreasing, which is the main cause of fruit senescence [46]. The decrease in the titratable acid content of the BITC-GSA and EUG-GSA films significantly slowed, with EUG-GSA showing the highest titratable acid content at the end of storage (1.3 g/100 g). The slow rate of decrease in the amount of titratable acid in cherries wrapped with BITC-GSA and EUG-GSA films could be attributed to the effective isolation of oxygen by the composite film, which slowed the respiration of the cherries, reduced the conversion rate of organic acids, and better preserved the flavor of the cherries.

The temporal changes in cherry pH across different treatment groups during storage are shown in Figure 5f. A universal increase in pH was observed in all groups over time. After 15 days, the control, PE, and GSA groups reached pH values of 5.7, 5.8, and 5.3, respectively, whereas the BITC-GSA and EUG-GSA groups demonstrated significantly slower increases in pH, attaining final values of only 4.8 and 4.6, respectively. When cherries are contaminated with microorganisms, the pH decreases as microbial growth and metabolic activity lead to the production of organic acids. GSA films incorporated with BITC and EUG effectively inhibited microbial proliferation, thereby helping to maintain the pH stability of the cherries.

As shown in Figure 5i, cherries in all the groups gradually experienced surface shrinkage, browning, and decay during storage. The control and PE groups exhibited more obvious deterioration, particularly at the later storage stages, whereas the BITC-GSA and EUG-GSA treatments better maintained the appearance of cherries. The EUG-GSA group showed the least visible decay and better surface integrity, which was consistent with the lower decay rate and improved quality retention observed in the quantitative results.

PCA and correlation analyses were used to comprehensively evaluate changes in cherry quality during storage. As shown in Figure 5g, PC1 and PC2 explained 87.8% and 8.4% of the total variance, respectively. In the PCA score plot, samples from the control and PE groups tended to shift toward the quality deterioration region during storage, whereas BITC-GSA and EUG-GSA samples were relatively closer to the region associated with quality maintenance, suggesting that the active films slowed the overall deterioration of cherries. The loading plot in Figure 5h shows that hardness, soluble solids content, and titratable acidity were associated with better quality maintenance, whereas the decay rate and pH were related to quality deterioration. Correlation analysis further supported these results (Figure 5j), showing that the hardness, soluble solids content, and titratable acidity were positively correlated with each other and negatively correlated with the decay rate and pH, whereas the decay rate was positively correlated with the pH and weight loss rate. These results, together with the visual appearance changes shown in Figure 5i, confirmed that the active films helped delay cherry deterioration during storage.

### 3.10. Preservation of Beef Freshness by BITC-GSA and EUG-GSA Films

**Figure 6:**
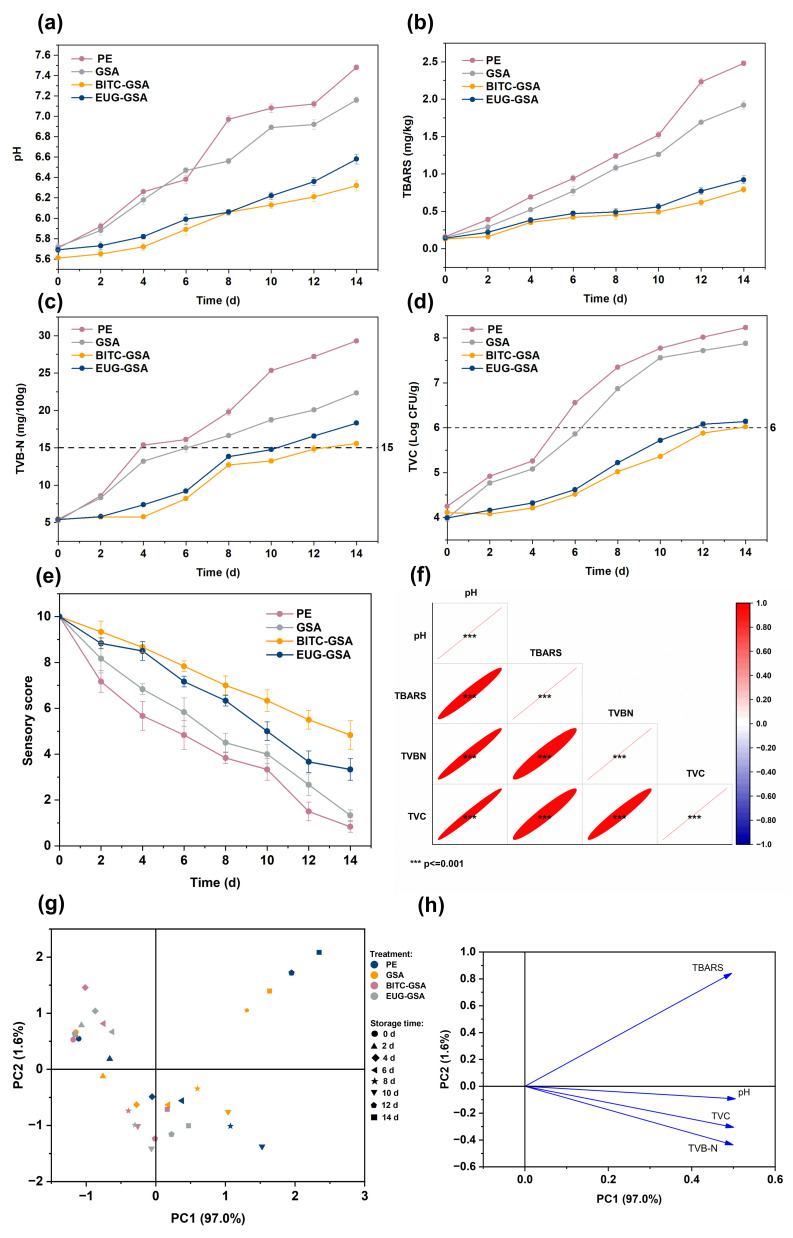
Effects of different films on beef preservation: (**a**) pH; (**b**) TBARS; (**c**) TVB-N; (**d**) TVC; (**e**) sensory evaluation; (**f**) correlation heatmap of beef quality indicators; (**g**) PCA loading plot; (**h**) PCA score plot.

The pH value is among the most important indicators of beef quality and is used to determine the degree of freshness or deterioration of beef [47]. Fresh meat usually has a pH less than 6 [48]. As shown in Figure 6a, during the initial period of storage (day 0–day 2), the pH value of the beef remained between 5.6 and 6.0 in all the treatment groups. The pH of beef wrapped with PE film and GSA film significantly increased to 6.3 and 6.2, respectively, on day 2 of storage and reached more than 7 on day 14. However, beef wrapped with BITC-GSA and EUG-GSA films remained at a lower pH during storage, reaching 6.3 and 6.5, respectively, on day 14 of storage. The increase in the pH of meat products during storage is due mainly to the activity of some microorganisms that produce alkaline compounds. The BITC-GSA and EUG-GSA films strongly inhibited the increase in the pH of the beef. This may be because BITC and EUG inhibited the propagation of alkaline compound-producing microorganisms in beef and effectively maintained the pH stability of beef during storage.

The concentration of TBARS increases when oxidation of lipids in beef occurs. In general, the recommended allowable level of TBARS in meat is approximately 2 mg/kg [49]. As shown in Figure 6b, the TBARS values of the PE and GSA film groups increased from 0.2 mg/kg to 2.5 mg/kg and 2.0 mg/kg, respectively, within 14 days. The TBARS values of beef wrapped with BITC-GSA and EUG-GSA films were 0.8 mg/kg and 0.9 mg/kg, respectively, on the 14th day, which were significantly lower than those of the PE film and GSA film groups. These findings suggest that the GSA films containing BITC and EUG have a certain degree of antioxidant activity, thus delaying the oxidation of lipids in beef to a certain extent.

During spoilage, beef produces organic acids, which combine with alkaline nitrogen-containing substances, such as ammonia and amines, produced by protein breakdown, to form volatile, saline nitrogen. Therefore, the degree of spoilage of beef can be determined by measuring the change in total volatile nitrogen. In accordance with the national standard (Chinese National Standard GB 2707-2016) [50], the content of TVB-N in meat should not exceed 15 mg/100 g. As shown in Figure 6c, during the storage period, the TVB-N value tended to increase with increasing magnitude. The TVB-N value of the PE film group (16.1 mg/100 g) exceeded 15 mg/100 g on the 6th day. In contrast, the BITC-GSA and EUG-GSA film groups maintained the standard of Grade 1 fresh meat for 10 days. This may be because the antimicrobial properties of BITC and EUG slowed the decomposition of proteins and amino acids by microbial contamination, which effectively retarded the spoilage of beef.

Spoilage of meat is closely associated with microbiological contamination. Therefore, the total viable count (TVC) is a key indicator for determining the spoilage status of meat. The change in the TVC of beef during refrigeration is shown in Figure 6d. A TVC value of 6 log CFU/g indicates that the meat has deteriorated [51]. The initial TVC values of the beef groups ranged from 4.0 to 4.3 log CFU/g. Throughout the storage period, the TVC values of all the groups gradually increased, with the TVC values of the PE and GSA films exceeding the permissible range of 6 log CFU/g on days 6 and 8, respectively. The TVC values of the BITC-GSA and EUG-GSA films reached 6.0 log CFU/g and 6.1 log CFU/g on the 14th and 12th days, respectively. Compared with PE films, BITC-GSA and EUG-GSA films prolong the shelf life of beef by 6–8 days. These results can be attributed to the good antimicrobial activity of BITC and EUG.

Sensory evaluation was conducted to comprehensively assess the acceptability of the beef samples during refrigerated storage, including color, odor, texture, viscosity, and visible impurities (Figure 6e). The sensory scores of all the groups gradually decreased with increasing storage time, indicating progressive changes in the sensory quality of the beef. Compared with the PE and GSA groups, the BITC-GSA and EUG-GSA groups maintained higher sensory scores, suggesting that the active films helped delay undesirable changes in odor, surface stickiness, color, and texture. In particular, the films containing BITC or EUG did not result in unacceptable sensory changes in beef under the tested conditions.

Correlation analysis further revealed significant positive correlations among pH, TBARS, TVB-N, and TVC (Figure 6f), indicating that these spoilage-related indicators were closely associated during refrigerated storage. PCA was then used to visualize the overall distribution of beef samples on the basis of these quality indicators. In the PCA score plot (Figure 6g), the PE and GSA samples at later storage stages were more clearly separated from the initial samples along PC1, whereas the BITC-GSA and EUG-GSA samples showed relatively smaller shifts, suggesting that the active films slowed the overall changes in beef quality. The loading plot (Figure 6h) revealed that pH, TBARS, TVB-N, and TVC contributed positively to PC1, indicating that PC1 reflected mainly changes associated with microbial growth, lipid oxidation, protein degradation, and pH variation. Overall, these results indicate that BITC-GSA and EUG-GSA films improved the preservation performance of beef during refrigerated storage.

## 4. Conclusions

This study demonstrated that BITC and EUG improved the overall performance of GSA-based active films. Compared with the pure GSA film, BITC-GSA and EUG-GSA showed higher tensile strength, improved water resistance, reduced water vapor permeability, and enhanced visible-light barrier properties. Notably, the water vapor permeability decreased by 65.4% and 59.2% after the incorporation of BITC and EUG, respectively, indicating improved barrier performance. SEM, FTIR, XRD, and TGA analyses further suggested that BITC and EUG affected the surface morphology, molecular interactions, internal structure, and thermal degradation behavior of the films. Application tests on cherries and beef revealed that both active films delayed quality deterioration during storage, as supported by physicochemical indicators, sensory/visual evaluation, PCA, and correlation analysis. Nevertheless, further studies are still needed to optimize the concentration of active compounds and systematically evaluate their release behavior, migration safety, long-term safety, and sensory acceptability under practical food packaging conditions, which will help support the broader application of these films.

## Figures and Tables

**Table 1:** Effects of the addition and non-addition of BITC and EUG on the color and opacity of GSA films.

Film	*L* *	a*	b*	Opacity (600_nm_/mm)	Photos
GSA	77.66 ± 3.24 ^a^	−3.42 ± 0.24 ^c^	4.43 ± 0.24 ^b^	1.85 ± 0.029 ^c^	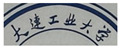
BITC-GSA	59.1 ± 0.88 ^b^	−0.76 ± 0.19 ^a^	5.09 ± 0.64 ^b^	2.64 ± 0.176 ^b^	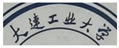
EUG-GSA	44.03 ± 0.26 ^c^	−1.45 ± 0.05 ^b^	17.79 ± 0.72 ^a^	2.86 ± 0.047 ^a^	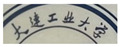

Values with different letters in a column indicate statistical significance at *p* < 0.05.

## Data Availability

The original contributions presented in this study are included in the article. Further inquiries can be directed to the corresponding author.

## Abbreviations

The following abbreviations are used in this manuscript:

G	Gelatin
SA	Sodium Alginate
BITC	Benzyl isothiocyanate
EUG	Eugenol
SEM	Scanning Electron Microscopy
FTIR	Fourier Transformed Infrared spectroscopy
XRD	X-ray Diffraction
TGA	Thermogravimetric Analysis
TS	Tensile Strength
EAB	Elongation At Break
WVP	Water Vapor Permeability
WCA	Water Contact Angle
PCA	Principal component analysis
